# Development and Validation of the Low Sit–High Step Test for Assessing Lower-Extremity Function in Sarcopenia

**DOI:** 10.3390/diagnostics16030480

**Published:** 2026-02-04

**Authors:** Serpil Demir, Burak Elçin, Ramazan Mert, İbrahim Kök, Onur Öz, Ethem Kavukçu, Nilüfer Balcı

**Affiliations:** 1 Department of Physical Medicine and Rehabilitation, Faculty of Medicine, Akdeniz University, Antalya 07070, Turkey; burakelcin97@gmail.com (B.E.); mert-rambo@outlook.com (R.M.); dribrahimkok@gmail.com (İ.K.); onuroz@akdeniz.edu.tr (O.Ö.); nilbalci@hotmail.com (N.B.); 2 Department of Sports Medicine, Faculty of Medicine, Akdeniz University, Antalya 07070, Turkey; ethemkavukcu@akdeniz.edu.tr

**Keywords:** low sit–high step test, sarcopenia, lower extremity, physical performance, muscle strength, geriatric assessment

## Abstract

**Objectives:** This study aimed to evaluate the validity, reliability, and diagnostic accuracy of the Low Sit–High Step (LS–HS) Test as an original, cost-effective, and clinically practical tool for assessing lower-extremity muscle strength and function, with a specific focus on its sensitivity in detecting early-stage sarcopenia. **Methods:** This cross-sectional study included 205 participants divided into four groups: probable sarcopenia, sarcopenia, and two control groups (young and middle-to-older adults). The LS–HS Test was compared across groups and against standard assessments to evaluate its efficacy in measuring lower-extremity function. Reliability was verified through Cronbach’s alpha and ICC. Multinomial logistic regression was used to determine the test’s predictive power, while ROC analysis assessed its diagnostic accuracy for sarcopenia screening. **Results:** The LS–HS scores were significantly higher in participants with probable sarcopenia and sarcopenia (*p*< 0.05). Multinomial logistic regression revealed that the LS–HS performance was a significant predictor of both probable sarcopenia and sarcopenia (*p* < 0.001). The test demonstrated excellent internal consistency (Cronbach’s α = 0.938) and very high inter-rater and test–retest reliability (ICC = 0.998). ROC analysis confirmed high diagnostic accuracy in distinguishing both probable sarcopenia (AUC = 0.768) and sarcopenia (AUC = 0.704) (all *p*< 0.01). **Conclusions:** The LS–HS Test is a valid, reliable, and sensitive tool for assessing lower-extremity functional capacity. Its ability to identify early functional decline, often manifesting before significant muscle mass loss, positions it as an effective alternative to traditional assessments in routine clinical practice, particularly for the early detection and monitoring of the sarcopenia spectrum.

## 1. Introduction

Sarcopenia is a disease characterized by the progressive loss of muscle mass and function that becomes increasingly prevalent with aging. It represents a serious health problem leading to functional impairments, reduced mobility, and an elevated risk of falls [1]. Historically, the diagnosis focused primarily on the quantitative loss of muscle mass; however, international consensus has shifted this paradigm to prioritize muscle function and strength. Recent diagnostic protocols now identify low muscle strength as the primary indicator of probable sarcopenia [2], emphasizing the concurrent assessment of strength and physical performance during early screening [3]. This hierarchical shift in consensus underscores the critical importance of detecting functional decline in its early stages before the structural loss becomes irreversible [1].

While handgrip strength (HGS) is traditionally utilized as the primary indicator of overall muscle strength, lower-extremity muscle strength serves as a far more critical determinant of functional independence in older adults. This is because the earliest and most pronounced age-related strength loss typically occurs in the proximal muscles of the lower limbs [4,5,6]. Despite its established role as a key measure of upper-body strength, HGS has limited capacity to represent lower-extremity power [4,7,8]. This diagnostic gap raises the concern that patients in the early stages of sarcopenia may be overlooked, highlighting the urgent need for more effective methods, such as the LS–HS Test, specifically designed to assess lower-limb functional capacity.

Although the Chair Stand Test (CST) is widely endorsed by current guidelines as a pragmatic alternative to the HGS for assessing muscle power, it often lacks the sensitivity required to detect the early, subtle stages of functional decline [9,10]. Emerging evidence suggests that HGS and CST are not interchangeable metrics; in fact, the CST frequently reports a lower prevalence of sarcopenia than HGS, potentially creating a false sense of security regarding a patient’s actual strength [10,11]. This discrepancy likely stems from a ceiling effect in standard assessments because the physical demand of the test, typically performed at standard chair heights, does not sufficiently challenge the individual’s functional reserve [12,13].

Our clinical observations support this concern, as many older adults who navigate standard-height chairs without difficulty experience significant struggle when faced with lower seating or higher steps. In such cases, individuals frequently rely on momentum-transfer strategies or compensatory maneuvers, such as pushing off their knees, to complete the movement [14]. While previous research has demonstrated a positive association between maximum step height and lower-limb power [15], a standardized and clinically accessible tool to measure this specific mechanical threshold directly has been missing from the literature.

Consequently, the Low Sit–High Step (LS–HS) test was developed to neutralize these compensatory strategies by increasing the biomechanical demand, thereby offering a more precise evaluation of lower-limb strength in alignment with modern diagnostic paradigms. The LS–HS Test was designed to fill this methodological gap by identifying early-stage functional losses that standard assessments may overlook. By challenging the lower-limb muscles at their most disadvantaged biomechanical angles and moment arms, the test provides a more rigorous evaluation than traditional metrics. Furthermore, the use of fixed platform heights ensures clinical standardization while neutralizing anthropometric variations through the equilibrium of joint torque requirements, thereby establishing an objective threshold for functional capacity. Accordingly, this study aimed to develop the LS–HS Test as a practical and cost-effective tool and to demonstrate its clinical utility, validity, and reliability in identifying functional deficits associated with sarcopenia.

## 2. Materials and Methods

### 2.1. Design

In this cross-sectional study, we aimed to develop a cost-effective, simple, and easily applicable test to assess lower-extremity muscle strength and aid in diagnosing sarcopenia.

### 2.2. Setting

The study was conducted at the Akdeniz University Hospital, Physical Medicine and Rehabilitation Clinic, over a 11month period from November 2022 to September 2023. Participants were recruited based on predefined inclusion and exclusion criteria. To ensure data consistency and minimize performance variability, the entire assessment protocol for each participant was completed within a single session on the same day. During this initial session, two researchers independently and blindly evaluated the LS–HS Test to determine inter-rater reliability. To evaluate test–retest reliability, participants returned to the measurement site for a second visit two weeks after the initial assessment, where the test was repeated by the same primary researcher. This two-week interval was selected to minimize potential learning effects while ensuring that no significant changes in functional status occurred.

### 2.3. Participants

A total of 217 individuals aged 18 to 85 years were initially enrolled in this cross-sectional study. Twelve participants were excluded due to noncompliance with the two-week follow-up protocol, leaving a final sample of 205 participants (151 women and 54 men) who completed all study procedures. Prior to inclusion, all participants underwent a comprehensive physical examination and medical history screening by a physician to ensure safety and diagnostic accuracy.

Inclusion Criteria:

1. Individuals aged between 18 and 85 years.
2. Ability to follow verbal instructions and maintain effective communication.
3. Ability to perform basic functional tasks independently.
4. Voluntary participation with a signed informed consent form.

Exclusion Criteria:

1. Hand and Upper Extremity Conditions: severe hand deformities, advanced osteoarthritis of the hand, or any neurological/orthopedic conditions (e.g., carpal tunnel syndrome, severe contractures) that significantly interfere with HGS.
2. Lower-Extremity and Mobility Conditions: severe joint deformities (e.g., advanced knee or hip osteoarthritis), acute spinal disorders, or a history of recent lower-limb surgery that prevents the safe execution of the sit-to-stand and step-up tasks.
3. Pain Factors: significant lower-extremity pain that could confound functional performance and result in biased test outcomes.
4. Secondary Sarcopenia: conditions potentially causing secondary sarcopenia, such as active malignancies, inflammatory rheumatic diseases, or severe neurological and endocrine disorders.
5. Physical Impairments: limb amputations or severe orthopedic impairments preventing the execution of the study protocol.

All participants were evaluated for sarcopenia according to the updated diagnostic criteria of the EWGSOP2 [2].

According to these criteria, in women, HGS below 16 kg indicated probable sarcopenia (low muscle strength). When low muscle strength was accompanied by an appendicular skeletal muscle mass (ASMM) below 15 kg, the diagnosis was classified as sarcopenia (low muscle quantity). If both low muscle strength and low muscle mass were present together with a gait speed of 0.8 m per second or lower, the condition was defined as severe sarcopenia (low physical performance). In men, an HGS below 27 kg indicated probable sarcopenia, an ASMM below 20 kg confirmed sarcopenia, and a gait speed of 0.8 m per second or lower indicated severe sarcopenia.

Based on these criteria, 33 participants aged 40 to 85 years were diagnosed with probable sarcopenia, and 23 participants were diagnosed with sarcopenia. Healthy individuals aged 18 to 39 years were assigned to the young control group, while healthy individuals aged 40 to 85 years without sarcopenia formed the middle-to-older control group.

The control groups were not preselected; rather, they consisted of non-sarcopenic individuals identified within the same study population according to the EWGSOP2 framework.

It is important to note that the LS–HS Test protocol assigns the maximum time score to participants who are unable to complete a task, ensuring that functional incapacity is documented without requiring the participant to exceed their safe physical limits.

### 2.4. Measurements

All participants were measured for HGS, ASMM, and gait speed, and the CST, Timed Up and Go (TUG) test, and LS–HS Test were administered to them.

**HGS:** This was measured using the Jamar hand dynamometer (Lafayette Instrument, Lafayette, IN, USA). Participants were seated in a chair with back support and their feet placed flat on the floor. The shoulders were held in a slightly adducted position, the elbow was flexed at 90°, and the forearm was maintained in a neutral position. Two measurements were taken from the dominant hand, and the mean value was used for analysis.

**ASMM:** This was determined by bioelectrical impedance analysis (BIA) using the Tanita Body Composition Analyzer (model BC-418, Tanita Corp, Tokyo, Japan). The BIA method was selected for its non-invasive nature and high reliability. Recent validation studies, such as Huang et al. (2025), have confirmed that Tanita BIA devices show high correlation (r > 0.90) with dual-energy X-ray absorptiometry (DXA) for assessing muscle mass in adult populations [16]. BIA was measured at the initial visit.

Assessments were performed in the morning following at least 4 h of fasting, provided that no strenuous exercise had been performed within the preceding 24 h. Participants were required to void their bladder and remove all metal accessories prior to the measurement. All measurements were conducted in a standing position. ASMM was calculated as the sum of muscle mass in both arms and legs.

**Gait speed:** Measured as the time required to walk 4 m at a usual pace using a stopwatch.

**CST:** Recorded as the time taken to stand up five times from a seated position without using the arms.

**TUG:** Measured as the time taken to rise from a standard chair, walk 3 m, turn, return, and sit down again.

**LS–HS Test:**

**Administration Standards and Safety:** The LS–HS Test was administered under standardized conditions by two researchers who are residents in physical medicine and rehabilitation, both experienced in functional clinical assessments. To ensure safety, a researcher remained within arm’s reach throughout the test to provide physical support if necessary. Prior to the study, both evaluators underwent comprehensive training to standardize the administration steps and minimize procedural variance. All participants performed the LS–HS Test under standardized conditions. To prioritize participant safety, a researcher remained within arm’s reach throughout the test to provide physical support if necessary.

The test protocol consisted of four sequential movements: performing a sit-to-stand task from a 20 cm stool, followed by stepping up and down from a 40 cm platform. To assess inter-rater reliability, two independent raters simultaneously and blindly recorded the participants’ performances during the initial session. To evaluate test–retest reliability, the same standardized procedure was repeated by a single researcher two weeks later. This consistent protocol was strictly applied to all participants to ensure procedural uniformity and minimize measurement bias.

**Test Components and Biomechanical Basis:** The test consists of four sequential movements utilizing a 20 cm stool for sitting tasks and a 40 cm platform for stepping tasks, the latter representing the average below-knee height (Figure S1). Participants are required to step up and down the 40 cm platform to assess lower-limb power and balance, and to perform a sit-to-stand task from the 20 cm stool to evaluate functional strength at deep joint angles.

(1) **40 cm Step-Up**—assesses concentric lower-limb strength, particularly of the hip and knee extensors, as well as balance and coordination.
(2) **40 cm Step-Down**—evaluates eccentric control of the quadriceps and balance during descent.
(3) **20 cm Sit-Down**—tests eccentric control and postural stability, simulating sitting onto low surfaces.
(4) **20 cm Stand-Up**—assesses concentric strength and power of the quadriceps and gluteal muscles, reflecting functional independence and fall risk.

**Graded Administration and Scoring:** A hierarchical approach was used to determine each participant’s “maximum independent capacity”:

**Procedure:** Participants were initially asked to perform the movement without specific instructions regarding support. If a participant used assistance (e.g., pushing on the knee), they were then encouraged to attempt the task at a more challenging (independent) level. The highest level of independence successfully demonstrated was recorded.

Scoring System (0–3 per item):

**0:** performs independently without support.
**1:** performs by pushing on their own knee (self-support).
**2:** performs using external support (researcher or object).
**3:** unable to perform the movement.
Total Score: results range from 0 (best) to 12 (worst).

### 2.5. Reliability and Bias Control

Inter-Rater Reliability: Two independent raters, blinded to group classifications and other clinical data, recorded performances simultaneously and independently during the initial session. Test–Retest Reliability: The same standardized procedure was repeated by a single researcher two weeks later. To minimize bias, evaluators remained blinded to participants’ sarcopenia diagnosis and all other measurement results throughout the process.

### 2.6. Statistical Methods

A priori power analysis was performed using G*Power 3.1 software to determine the minimum required sample size for the statistical analyses. Since no prior studies have evaluated the LS-HS Test, the expected effect size was estimated based on Cohen’s conventional criteria for behavioral and clinical research. Assuming a medium effect size (f^2^ = 0.25), a significance level (α) of 0.05, and a statistical power (1–β) of 0.80, the required sample size for a multiple linear regression model with five predictors (age, BMI, HGS, CST, and TUG) was calculated as 128 participants [17].

The final study sample of 205 participants exceeded this requirement, indicating that the study had sufficient statistical power to detect meaningful associations. Therefore, the analyses conducted in this study are considered adequately powered to support the validity and reliability findings of the LS-HS Test.

Statistical analyses were conducted using IBM SPSS Statistics for Windows, Version 23.0 (IBM Corp., Armonk, NY, USA). A *p*-value < 0.05 was considered statistically significant.

Descriptive statistics were presented as mean ± standard deviation (SD) for normally distributed continuous variables and as median (Q1–Q3) for non-normally distributed variables. The normality of data distribution was tested using both the Shapiro–Wilk and Kolmogorov–Smirnov tests.

For group comparisons, parametric tests (independent-sample *t*-test or one-way ANOVA with Tukey’s HSD post hoc analysis) were applied when the normality and homogeneity of variance assumptions were met. Tukey’s HSD was specifically chosen because it provides robust control of the Type I error rate when variances are equal and group sizes are approximately balanced. Homogeneity of variances was confirmed using Levene’s test (*p* > 0.05). When these assumptions were violated, non-parametric tests (Mann–Whitney U or Kruskal–Wallis test with Bonferroni-adjusted post hoc comparisons) were used.

Categorical variables were expressed as counts (*n*) and percentages (%), and compared using the chi-square test. A *p*-value < 0.05 was considered statistically significant.

### 2.7. Reliability Analysis

The reliability and validity of the LS-HS Test were comprehensively evaluated.

Internal consistency was assessed using Cronbach’s alpha (α) to determine the homogeneity of the four test items (40 cm step-up, 40 cm step-down, 20 cm sit-down, and 20 cm stand-up). Inter-rater and test–retest reliability were examined using a two-way random effects model with absolute agreement, and intraclass correlation coefficients (ICCs) were reported with 95% confidence intervals (CIs).

Construct validity was analyzed through exploratory factor analysis (EFA). Criterion validity was tested by correlating LS-HS Test scores with HGS, ASMM, gait speed, CST, and TUG results using Pearson or Spearman correlation analyses as appropriate.

Discriminant validity was evaluated by comparing groups using one-way ANOVA or Kruskal–Wallis tests with Bonferroni-adjusted post hoc analyses.

Predictive validity was assessed using linear and logistic regression analyses, and diagnostic accuracy was determined through ROC analysis, including the calculation of the AUC and Youden index.

### 2.8. Ethics Statement

This study protocol was approved by the Ethics Committee of Akdeniz University Faculty of Medicine (KAEK-637, 26 October 2022) and conducted in accordance with the ethical standards of the 2000 Declaration of Helsinki.

All participants provided written informed consent prior to participation.

This study adheres to the STROBE guidelines [18] and includes the checklist in Checklist S1.

## 3. Results

The average age of the 205 participants (151 females, 54 males) was 56.21 (14.00) (mean (SD); 18 to 84 years) (Table 1).

**Figure 1:**
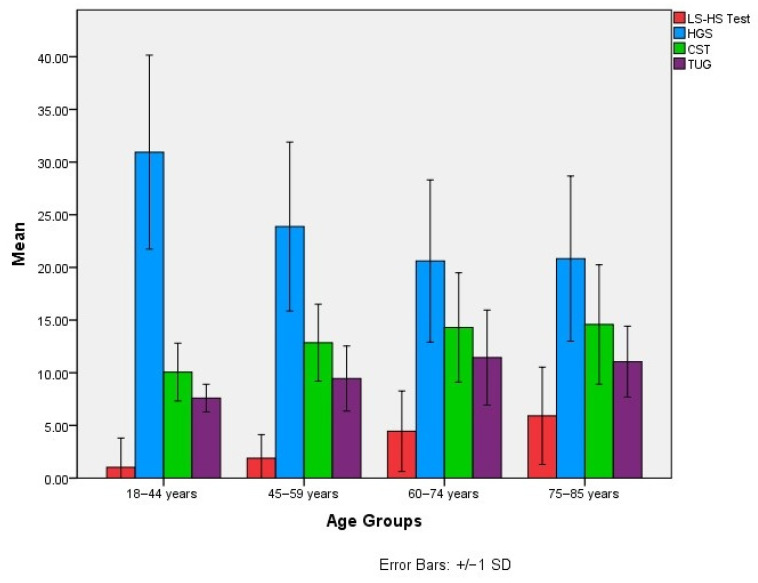
Comparison of physical performance tests across age groups. Bars represent the mean, and error bars represent the standard deviation (SD). LS–HS Test: Low Sit–High Step Test (score), HGS: Handgrip Strength (kg), CST: Chair Stand Test (sec), TUG: Timed Up and Go (sec).

When patients were divided into four age groups according to the World Health Organization classification, namely young adults (18–44 years), middle-aged adults (45–59 years), older adults (60–74 years), and elderly adults (75–84 years), HGS and ASMM values showed a gradual decline with advancing age. In contrast, LS-HS Test, CST, and TUG scores increased with age, reflecting decreased physical performance and functional capacity (Figure 1).

### 3.1. Reliability Analysis

**Internal Consistency:** The LS-HS Test demonstrated excellent internal reliability. Cronbach’s α was 0.938 and 0.940 when calculated based on standardized items, indicating excellent internal consistency across the four components of the test (step-up, step-down, sit-down, and stand-up). The mean scores of these items ranged from 0.61 (0.89) to 0.93 (1.05). Inter-item correlation coefficients ranged from 0.72 to 0.91, demonstrating strong positive relationships among the test items and confirming the homogeneity of the test content. To further assess reliability, an intraclass correlation coefficient (ICC) analysis was performed using a two-way random-effects model with absolute agreement. The single-measure ICC was 0.778 (95% CI: 0.728–0.821), and the average-measure ICC was 0.933 (95% CI: 0.914–0.948), both statistically significant (*p* < 0.001). These findings confirm that the LS-HS Test has excellent internal consistency and strong reliability across items, raters, and repeated measurements.

**Inter-Rater Reliability:** A total of 205 participants were evaluated independently by two raters. The inter-rater reliability of the LS-HS Test was analyzed using the intraclass correlation coefficient (ICC) based on a two-way random-effects model with absolute agreement. The single-measure ICC was 0.995 (95% CI: 0.993–0.996, *p* < 0.001), indicating very high agreement between individual scores obtained by different raters. The average-measure ICC was 0.997 (95% CI: 0.997–0.998, *p* < 0.001), confirming near-perfect reliability when the mean scores from multiple raters were considered. The F-test for reliability was also statistically significant (F (204, 204) = 386.93, *p* < 0.001), demonstrating that the LS-HS Test yields highly consistent results between different evaluators.

**Test–Retest Reliability:** To assess the stability of the LS-HS Test over time, 205 participants completed the test twice, with a two-week interval between sessions. The single-measure ICC was 0.995 (95% CI: 0.993–0.996, *p* < 0.001), showing strong agreement between the two time points, and the average-measure ICC was 0.998 (95% CI: 0.997–0.998, *p* < 0.001), confirming excellent temporal reliability. The F-test for reliability was statistically significant (F (198, 198) = 385.76, *p* < 0.001), indicating that the LS-HS Test demonstrates excellent repeatability and measurement stability over time.

### 3.2. Validity Analysis

**Construct Validity:** The construct validity of the LS-HS Test was evaluated using exploratory factor analysis (EFA) based on the four test items. The Kaiser–Meyer–Olkin (KMO) measure of sampling adequacy was 0.738, and Bartlett’s Test of Sphericity was statistically significant (p < 0.001), confirming that the data were suitable for factor analysis.

A single factor was extracted, explaining 84.78% of the total variance, with factor loadings ranging from 0.906 to 0.930. These results indicate that all four items measure a common underlying construct—lower-extremity muscle strength and function—thus supporting the unidimensional structure and strong construct validity of the LS-HS Test.

**Criterion Validity:** This parameter was examined by correlating LS-HS Test scores with established functional and physiological parameters. LS-HS scores showed positive correlations with age, BMI, CST, and TUG, and negative correlations with HGS, ASMM, and gait speed (all *p* < 0.05) (Table S1).

These findings confirm that higher LS-HS scores are associated with poorer muscle strength and physical performance, supporting the criterion validity of the test.

**Discriminant Validity**: When the four groups were compared, LS-HS Test scores were significantly higher in the probable sarcopenia and sarcopenia groups compared to both control groups (all *p*< 0.05) (Table 2). This finding indicates that the LS-HS Test successfully distinguishes between individuals with different levels of muscle strength and function.

A multiple linear regression model was constructed to examine the determinants of LS-HS Test scores using age, BMI, HGS, CST, and TUG as predictors. The overall regression model was statistically significant (F (8, 199) = 43.158, *p* < 0.001), explaining 52% of the variance in LS-HS scores (R^2^ = 0.520, adjusted R^2^ = 0.508). Among the predictors, age, BMI, HGS, CST, and TUG were identified as significant independent contributors to LS-HS performance (Table S2).

**Predictive Validity:** To evaluate the predictive validity of the LS-HS Test for identifying probable sarcopenia and sarcopenia, a multinomial logistic regression analysis was performed, including LS-HS Test scores, age, BMI, and sex as predictors. The model was statistically significant (χ^2^ (8) = 71.311, *p* < 0.001), indicating that the predictors effectively differentiated among the outcome categories. The model explained 29.4% (Cox & Snell R^2^), 37.4% (Nagelkerke R^2^), and 22.5% (McFadden R^2^) of the variance in the dependent variable, with a –2 Log Likelihood of 244.945 (Table 3).

These results demonstrate that the LS-HS Test, in combination with demographic and physical parameters, exhibits strong predictive capacity for identifying individuals at risk of sarcopenia.

**Figure 2:**
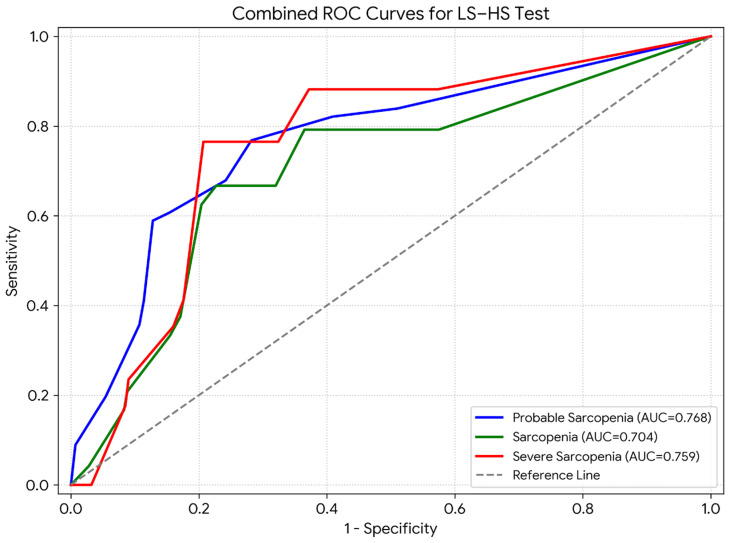
ROC curves for probable sarcopenia, sarcopenia, and severe sarcopenia.

**Diagnostic Accuracy:** Receiver operating characteristic (ROC) curve analysis was conducted to evaluate the diagnostic accuracy of the LS-HS Test in identifying probable sarcopenia, sarcopenia, and severe sarcopenia. The LS-HS Test demonstrated a significant ability to discriminate between different stages of sarcopenia (all *p* < 0.01) (Table 4, Table S3 and Figure 2). The classification performance metrics were as follows:

- Probable sarcopenia model: accuracy = 0.732 (73.2%), F1 score = 0.610 (61.0%);
- Sarcopenia model: accuracy = 0.761 (76.1%), F1 score = 0.395 (39.5%);
- Severe sarcopenia model: accuracy = 0.790 (79.0%), F1 score = 0.377 (37.7%).

These results indicate that the LS-HS Test achieves moderate-to-high accuracy in detecting the presence and severity of sarcopenia.

**Other Analyses:** When participants were categorized into healthy, probable sarcopenia, and sarcopenia groups according to our proposed the LS–HS Test cut-off scores (1–2 scores: probable sarcopenia; 3–12 scores: sarcopenia), significant differences were found in age, HGS, gait speed, and CST scores across all groups (*p* < 0.05; all *p* < 0.017 after Bonferroni correction). In contrast, ASMM and TUG scores were similar between healthy and probable sarcopenia individuals but significantly differed in the sarcopenia group compared to the other two groups (Table S4).

These findings suggest that the LS-HS Test can accurately classify individuals across the sarcopenia spectrum and is sensitive to clinically relevant functional differences.

**LS–HS Test Performance and Observations:**

No participants, including those in the sarcopenia and probable sarcopenia groups, reported new-onset or aggravated spinal or joint pain; furthermore, no injuries or adverse events were recorded during or after the administration of the LS–HS Test.

During the LS–HS test administration protocol, it was observed that the vast majority of participants initially initiated the movement using the strategy most suited to their perceived functional capacity. Among those who initially started the task by pushing off their knees (Score 1), only three participants were able to complete the movement independently (Score 0) following the researcher’s guidance. When participants who initially required external support (Score 2) were asked to attempt the movement using only two self-supports (pushing off their knees), only a few participants were successful; all other participants in this group failed at this scoring level.

## 4. Discussion

This study aimed to evaluate the validity and reliability of the LS–HS Test as a practical measure of lower-limb muscle strength and function. Our findings confirmed that the LS–HS Test is a valid, reliable, and clinically applicable tool. Furthermore, the LS–HS Test performance demonstrated significant associations with age, HGS, gait speed, CST, and TUG results, reinforcing its validity as a clinical assessment tool. Contrary to potential concerns regarding secular trends, our findings showed a clear and expected differentiation between age groups. Younger participants exhibited significantly better performance in CST, TUG, and LS-HS tests, as well as higher gait speeds, compared to the middle-aged group, confirming that age-related functional changes were clearly captured. Additionally, even after adjusting for age, gender, and BMI, LS–HS scores remained independently associated with sarcopenia. Moreover, the ROC analysis results validated the diagnostic value of the test by exhibiting high sensitivity and specificity in identifying individuals with sarcopenia.

Research indicates that muscle strength declines linearly at a rate of approximately 1.2–1.3% per year from the third decade of life, with a cumulative loss of roughly 60% by age 85 [11,19,20]. While age was associated with sarcopenia, the LS–HS Test remained significantly correlated with both probable sarcopenia and sarcopenia. This suggests the test captures sarcopenia-specific functional decline beyond physiological aging. In this context, while HGS is a common proxy for overall strength, it often fails to reflect lower-limb capacity [4,7,8]. With age, knee extensor strength declines more rapidly than HGS; since knee extensors are critical for standing and walking, their direct assessment is essential for early detection [4,5,6,12,21].

The CST, specifically performed as the 5-Times Sit-to-Stand (5STS) protocol, is widely recognized as a fundamental measure of lower-limb muscle power and a key component of physical performance batteries [9,10]. Endorsed by the EWGSOP2 as a primary alternative to HGS, the CST serves as a simple and accessible functional measure of lower-extremity strength and performance [2,12,22,23,24]. However, it has been suggested that HGS and CST are not equivalent indicators of muscle strength, with the CST often underestimating sarcopenia prevalence [11,12]. Furthermore, because the standard CST relies on the time taken at a standard chair height, individuals may utilize momentum, leading to an inherent ‘ceiling effect’ in higher-functioning individuals [13].

The LS–HS Test evaluates lower-extremity performance by strategically targeting functional points of maximal muscular disadvantage. Unlike traditional assessments, it is structured into two complementary phases. The low-sit phase (20 cm) mandates a transition from deep knee flexion (130–150°), forcing the musculature to operate where torque-generation capacity is most compromised. This exposes functional reserve deficits that often remain masked in tests with greater mechanical advantages. The high-step phase (40 cm) utilizes unilateral loading to demand maximal concentric and eccentric control. While fixed test surfaces (20 cm and 40 cm) might appear restrictive, they are grounded in methodological rationales. Biomechanically, although taller individuals benefit from wider knee angles, their longer limb segments inherently increase moment arms. This requires greater internal muscle torque, effectively neutralizing potential angular advantages [25]. Regarding body mass, higher weight often correlates with greater absolute strength due to chronic anti-gravity loading [26]. Thus, the test does not penalize individuals for their mass but provides an objective assessment of the power-to-weight ratio for functional independence [8].

By shifting the focus from speed to the mechanical threshold of strength, the LS–HS Test forces the musculoskeletal system to operate near its functional limits. This enables the identification of early and subtle declines that are frequently overlooked by current diagnostic criteria. In the study, the LS–HS Test demonstrated a stronger correlation with HGS than the CST. Furthermore, the LS–HS Test exhibited superior correlations with muscle mass (ASMM) and physical performance (indicators such as gait speed and TUG) compared to the CST [2,3,22]. This reflects an integrated functional capacity. This approach is supported by Nyberg et al. [15], who reported that maximal step-up height was strongly linked with knee extension strength. After controlling for age, BMI, and gender, the LS–HS Test retained strong predictive value. ROC curve analysis demonstrated a high ability to distinguish healthy individuals from those across the sarcopenia spectrum.

Current consensus guidelines prioritize low muscle strength as the primary diagnostic criterion [2,3], while reduced muscle mass is considered clinically significant only when associated with functional impairment [27,28]. The importance of this functional approach is supported by Beaudart et al. [29], who reported that poor muscle function is a more potent predictor of adverse outcomes than muscle mass alone. By focusing on the mechanical threshold of strength, the LS–HS Test aligns with these consensus-driven diagnostic priorities. The test serves as a comprehensive tool reflecting both strength and functional decline. Its relatively weak association with ASMM highlights that it is more sensitive to functional impairment than mere structural loss. This aligns with the call from GLIS for function-oriented assessment tools.

In the absence of a universally accepted diagnostic gold standard for sarcopenia, identifying functional declines in movements that healthy individuals can normally perform has become critically important. The LS–HS Test addresses these priorities by providing a practical and sensitive method for detecting early-stage impairment. Notably, our study revealed that LS–HS scores clearly differentiate healthy controls from individuals across the sarcopenia spectrum. However, the lack of a significant difference in test performance between the ‘probable sarcopenia’ and ‘confirmed sarcopenia’ groups suggests that these populations share a nearly identical functional impairment profile. This is a crucial finding, as current diagnostic frameworks distinguish confirmed from probable sarcopenia solely based on low muscle mass. Interestingly, when participants were grouped by the LS–HS cut-off values, muscle mass remained similar between healthy controls and the probable sarcopenia group, only declining significantly in the confirmed stage. These findings suggest that functional impairment, the principal driver of physical disability, becomes clinically evident well before muscle mass declines below established diagnostic thresholds. Ultimately, this evidence supports a shift in clinical focus from a purely mass-based diagnosis toward a strength-centered functional assessment, proving that the LS–HS Test is a highly sensitive tool for capturing early-stage decline often overlooked by traditional criteria.

Notably, our study revealed that LS–HS scores clearly differentiate healthy controls from individuals across the sarcopenia spectrum. However, the lack of a significant difference in test performance between the ‘probable sarcopenia’ and ‘confirmed sarcopenia’ groups suggests that these populations share a nearly identical functional impairment profile. This is a crucial finding, as current diagnostic frameworks distinguish confirmed from probable sarcopenia solely based on low muscle mass. Interestingly, when participants were grouped by LS–HS cut-off values, muscle mass remained similar between healthy controls and the probable sarcopenia group, only declining significantly in the confirmed stage. These results indicate that functional loss—the primary driver of physical disability—is already fully manifest before muscle mass falls below clinical thresholds. Ultimately, this evidence supports a shift in clinical focus from a purely mass-based diagnosis toward a strength-centered functional assessment, proving that the LS–HS Test is a highly sensitive tool for capturing early-stage decline often overlooked by traditional criteria.

The scoring system of the LS-HS test is designed as a hierarchical ordinal scale. Each of the four tasks is scored from 0 (complete independence) to 3 (functional failure). This 12-point cumulative approach allows clinicians to distinguish between ‘fit’ individuals and those who are beginning to compensate for muscle weakness by using their hands—an early indicator of pre-clinical disability that binary tests often fail to capture. It is worth noting that even a one-point variation in the score may signal early-stage functional impairment. To ensure clinical practicality, fixed heights of 20 cm and 40 cm were selected. This standardized approach ensures feasibility without requiring individual measurements. Stepping from a 40 cm platform requires a transition from knee flexion (<90°) to extension, while rising from a 20 cm stool necessitates extension from deep flexion (130°–150°). Higher surfaces pose significant challenges even for healthy subjects, whereas lower steps might result in a ‘ceiling effect’. Similarly, the 20 cm height exposes subtle deficits. Surfaces lower than 20 cm increase compressive loads on joints, posing safety risks, while higher stools lack sensitivity. This graded system provides a nuanced interpretation of frailty. Participants appeared aware of their limits, initiating the test at an appropriate level. This confirms the ecological validity of the LS–HS Test and its ease of comprehension.

All evaluations were conducted by an experienced practitioner. Rising and stepping are demanding yet familiar movements. This familiarity acted as an inherent safety mechanism; participants instinctively stopped the test if unstable. Consequently, no adverse events were recorded, suggesting the LS–HS Test is a safe clinical tool. Scores exhibited a physiological increase with age, consistent with neuromuscular decline. However, the test remains a powerful tool for identifying sarcopenia beyond chronological aging. This is not merely a reflection of aging, but a sensitive indicator of pathological decline.

This study has several limitations. First, its single-center, cross-sectional design restricts the evaluation of the LS–HS Test’s predictive capacity for sarcopenia progression; therefore, longitudinal and multicenter studies are needed to confirm its prognostic validity. Second, socioeconomic characteristics, specific medication protocols, and detailed physical activity levels were not recorded. While these factors can influence muscle performance, our objective was to develop a universal and practical tool for a broad population; thus, we believe these variables did not fundamentally alter the primary outcomes, as the test relies on universally feasible, fundamental movements. Although the sample size of the sarcopenia subgroup reflects the clinical prevalence inherent in a cross-sectional study, the large effect sizes observed between groups and the high explanatory power of the statistical models confirm that the results are robust. These findings indicate that the LS–HS Test possesses high sensitivity in detecting functional declines, even within a relatively small patient cohort. Importantly, future research incorporating longitudinal designs is required to track the progression of LS–HS scores over time, which would provide a more detailed understanding of its role in monitoring functional decline.

## 5. Conclusions

This study introduces the LS–HS Test as a practical, cost-effective, and highly sensitive tool for identifying functional deficits associated with sarcopenia. Our findings demonstrate that the test effectively distinguishes between healthy individuals and those across the sarcopenia spectrum, capturing age-related functional decline that traditional criteria might overlook. Notably, the clear performance differentiation observed between age groups confirms the test’s robustness against potential secular trends. By focusing on the mechanical threshold of strength through fundamental movements, the LS–HS Test serves as a realistic functional threshold for independence and a reliable measure of lower-limb power reserve. Its simplicity and minimal equipment requirements make it uniquely positioned for rapid implementation in diverse clinical settings. Future longitudinal research is encouraged to explore its prognostic value in monitoring disease progression and assessing the long-term efficacy of interventions.

## Figures and Tables

**Table 1:** Demographic and clinical characteristics of all participants.

		*n* (%) 205 (100%)	Mean (SD)	Median (Q1–Q3)
**Age (years)**			56.21 (14.00)	59 (49–66)
**Sex** (*n*/%)	**Female**	151 (73.7%)		
	**Male**	54 (26.3%)		
**BMI** (kg/m^2^)			27.77 (4.34)	27.60 (24.4–30.76)
**HGS** (kg)			23.53 (8.83)	22 (18–28)
**ASMM** (kg)			20.28 (4.20)	19.60 (17.2–22.6)
**Gait Speed** (m/s)			0.83 (0.26)	0.80 (0.67–0.96)
**CST** (s)			13.09 (4.60)	12.00 (10.00–15.35)
**TUG** (s)			10.07 (3.84)	9.00 (7.69–11.04)
**LS-HS Test** (score)			3.05 (3.57)	2 (0–6)
**Sarcopenia**	**None**	149 (72.7%)		
	**Probable Sarcopenia**	33 (16.1%)		
	**Sarcopenia**	7 (3.4%)		
	**Severe Sarcopenia**	16 (7.8%)		

BMI: Body Mass Index. HGS: Handgrip Strength. ASMM: Appendicular Skeletal Muscle Mass. CST: Chair Stand Test. TUG: Timed Up and Go Test. LS-HS Test: Low Sit–High Step Test.

**Table 2:** Comparison of anthropometric characteristics and physical performance test scores.

		Young Control Group (*n* = 27)	Middle-to-Older Control Group (*n* = 122)	Probable Sarcopenia Group (*n* = 33)	Sarcopenia Group (*n* = 23)	*p*	ε^2^
		Median (Q1–Q3)	Median (Q1–Q3)	Median (Q1–Q3)	Median (Q1–Q3)		
**Age** (years)		32 (25–35) ^a^	58 (50–65) ^bd^	66 (62–72) ^cd^	62 (57–73) ^bcd^	0.001 *	0.419
**Sex** *n* (%)	**Female**	18 (67%)	91 (75%)	23 (69.7%)	19 (82.6%)	0.538	
	**Male**	9 (33%)	31 (25%)	10 (30.3%)	4 (17.4%)		
**BMI** (kg/m^2^)		26.6 (23.4–29.3) ^abcd^	29.4 (25.2–30.9) ^abc^	28.3 (26.5–31.3) ^abc^	24.4 (23.1–26.2) ^ad^	0.001 *	0.078
**HGS** (kg)		28 (21–37) ^ab^	23 (20–30) ^ab^	15 (14–19) ^cd^	14 (11–15) ^cd^	0.001 *	0.461
**ASMM** (kg)		20.2 (17.5–23) ^abc^	20 (17.5–23) ^abc^	19.8 (18.2–22.7) ^abc^	14.8 (14.4–15) ^d^	0.001 *	0.205
**Gait Speed** (m/s)		0.9 (0.9–1.1) ^a^	0.8 (0.7–1) ^bd^	0.7 (0.8–0.8) ^cd^	0.7 (0.5–0.9) ^bcd^	0.001 *	0.157
**CST** (s)		9.6 (8–11.1) ^a^	11.9 (9.6–14) ^b^	15.8 (12.1–20.5) ^cd^	15 (12.3–19) ^cd^	0.001 *	0.236
**TUG** (s)		8.0 (6.8–8.5) ^a^	8.7 (7.5–10.2) ^b^	11.0 (9.5–14.1) ^cd^	11.4 (9.8–19) ^cd^	0.001 *	0.248
**LS-HS Test** (score)		0 (0–0) ^a^	1 (0–4) ^b^	6 (2–8) ^cd^	6 (3–8) ^cd^	0.001 *	0.240

*: *p* < 0.01. ε^2^: Epsilon squared. BMI: Body Mass Index. HGS: Handgrip Strength. ASMM: Appendicular Skeletal Muscle Mass. CST: Chair Stand Test. TUG: Timed Up and Go Test. LS-HS Test: Low Sit–High Step Test. Young Control Group (18–39 years). Middle-to-Older Control Group (40–85 years). Probable Sarcopenia Group (40–85 years). Sarcopenia Group: aged (40–85 years). Note: Data are presented as median (Q1–Q3). Statistical significance was determined using the Kruskal–Wallis test. *p* < 0.0125 was considered statistically significant after Bonferroni correction (0.05/4 groups) for post hoc pairwise comparisons. Different superscript letters (a, b, c, d) within the same row indicate statistically significant differences between the following groups: a: Young Control Group 1, b: Middle-to-Older Control Group 2, c: Probable Sarcopenia Group, d: Sarcopenia Group.

**Table 3:** Multinomial logistic regression analysis for predicting probable sarcopenia and confirmed sarcopenia.

Predictor	B	SE	Wald	*p*	Exp (B)	95% CI Lower	95% CI Upper
	**Category = 2 (Sarcopenia Group (** ***n*** **= 23)). Reference = 0 (Healthy Control (n = 149)**						
**İntercept**	−5.19	2.319	5.009	0.025 *			
**Age (years)**	0.067	0.024	7.775	0.005 **	1.069	1.02	1.121
**BMI (kg/m^2^)**	−0.024	0.055	0.183	0.669	0.977	0.877	1.088
**LS-HS Test (score)**	0.242	0.073	11.009	0.001 **	1.274	1.104	1.471
**Sex (1 = Female)**	−0.792	0.551	2.065	0.151	0.453	0.154	1.334
	**Category = 1 (Probable Sarcopenia Group (** ***n*** **= 33). Reference = 0 (Healthy Control Group (** ***n*** **= 149))**						
**Intercept**	1.03	2.388	0.186	0.666			
**Age (years)**	0.044	0.025	3.15	0.076	1.045	0.995	1.097
**BMI (kg/m^2^)**	−0.24	0.071	11.386	0.001 **	0.787	0.685	0.904
**LS-HS Test (score)**	0.308	0.084	13.46	0.001 **	1.36	1.154	1.603
**Sex (1 = Female)**	−0.315	0.71	0.197	0.657	0.73	0.182	2.931

*: *p* < 0.05. **: *p* < 0.001. Bold values indicate statistical significance at *p* < 0.01. LS-HS Test: Low Sit–High Step Test. Note: B: unstandardized coefficient; SE: standard error; Wald: Wald chi-square statistic; Exp(B): odds ratio; CI: confidence interval.

**Table 4:** ROC curve analysis for predicting probable sarcopenia, sarcopenia, and severe sarcopenia.

	AUC (SE)	*p*	95% CI	Cut-Off	Sensiv.	Spesif.	J
**Probable Sarcopenia**	0.768 (0.039)	0.001 *	0.691–0.844	0.5 ^a^	0.839	0.490	0.329
				2.5 ^b^	0.768	0.718	0.486
**Sarcopenia**	0.704 (0.059)	0.001 *	0.589–0.820	2.5 ^a^	0.792	0.635	0.427
				4.5 ^b^	0.667	0.773	0.440
**Severe Sarcopenia**	0.759 (0.057)	0.001 *	0.646–0.870	5.5 ^ab^	0.765	0.793	0.558

*: *p* < 0.01. ^a^: cut-off. ^b^: Our proposed cut-off. J: Youden’s Index. AUC: area under the curve. SE: standard error. CI: confidence interval.

## Data Availability

The data presented in this study are available upon request from the corresponding author due to the ethical requirement to protect participant confidentiality and comply with hospital data protection regulations.

## Supplementary Materials

The following supporting information can be downloaded at: https://www.mdpi.com/article/10.3390/diagnostics16030480/s1, Figure S1: Low Sit–High Step Test; Table S1: Correlation between LS–HS Test scores and clinical data; Table S2: Linear regression analysis for the LS–HS Test scores; Table S3: Diagnostic performance of the LS–HS Test at various cut-off scores; Table S4: Group comparisons based on LS–HS Test classification; Checklist S1: STROBE Statement checklist.

## Abbreviations

HGS	Hand Grip Strength:
ASMM	Appendicular Skeletal Muscle Mass
CST	Chair Stand Test
TUG	Timed Up and Go Test
LS–HS Test	Low Sit–High Step Test
